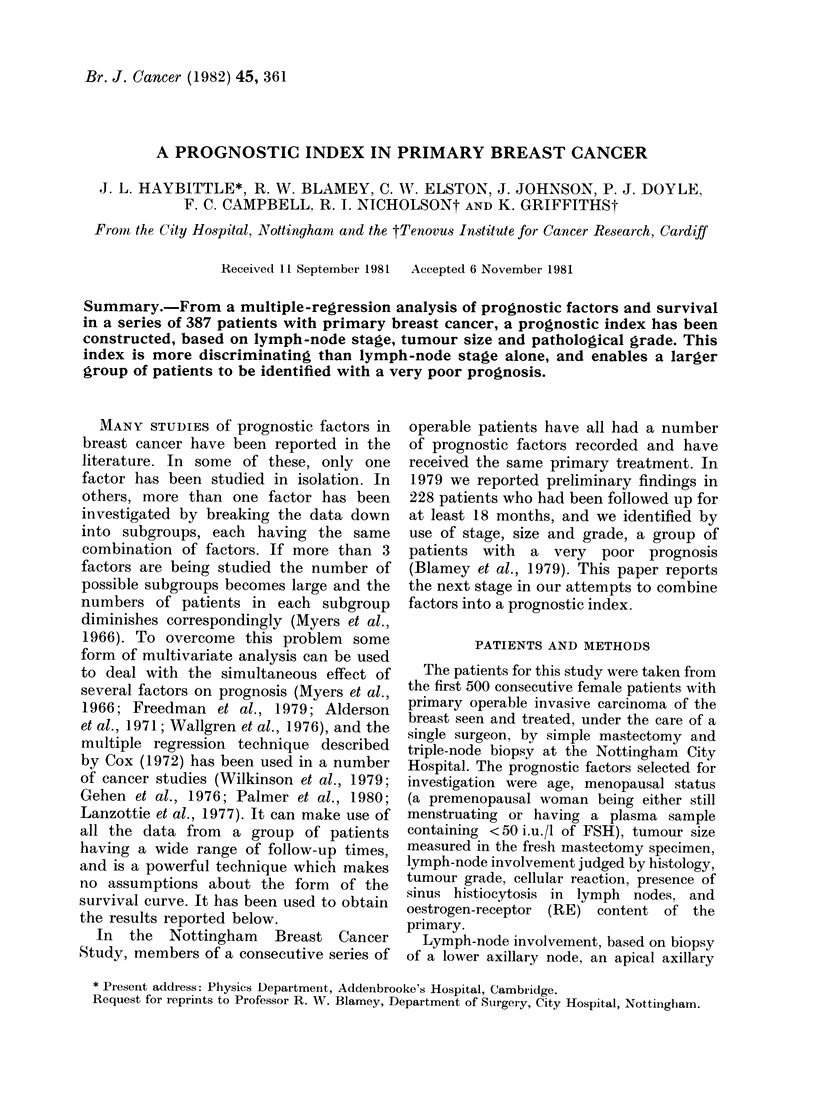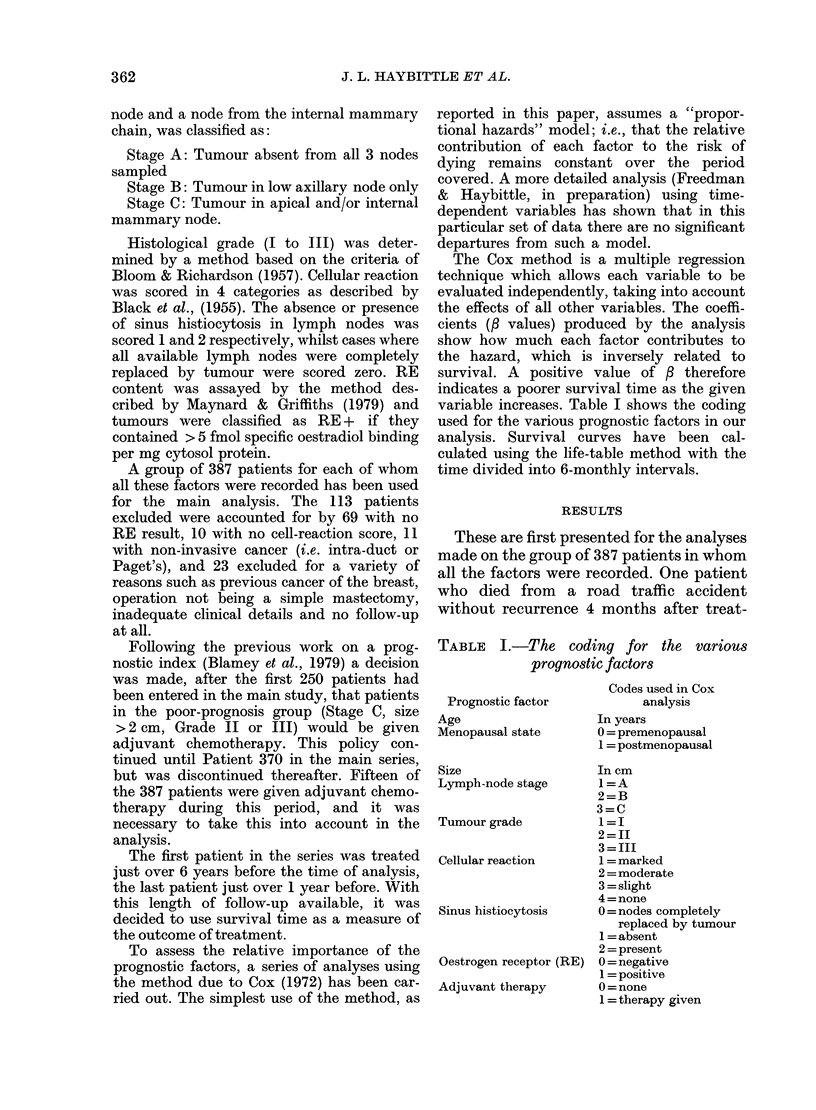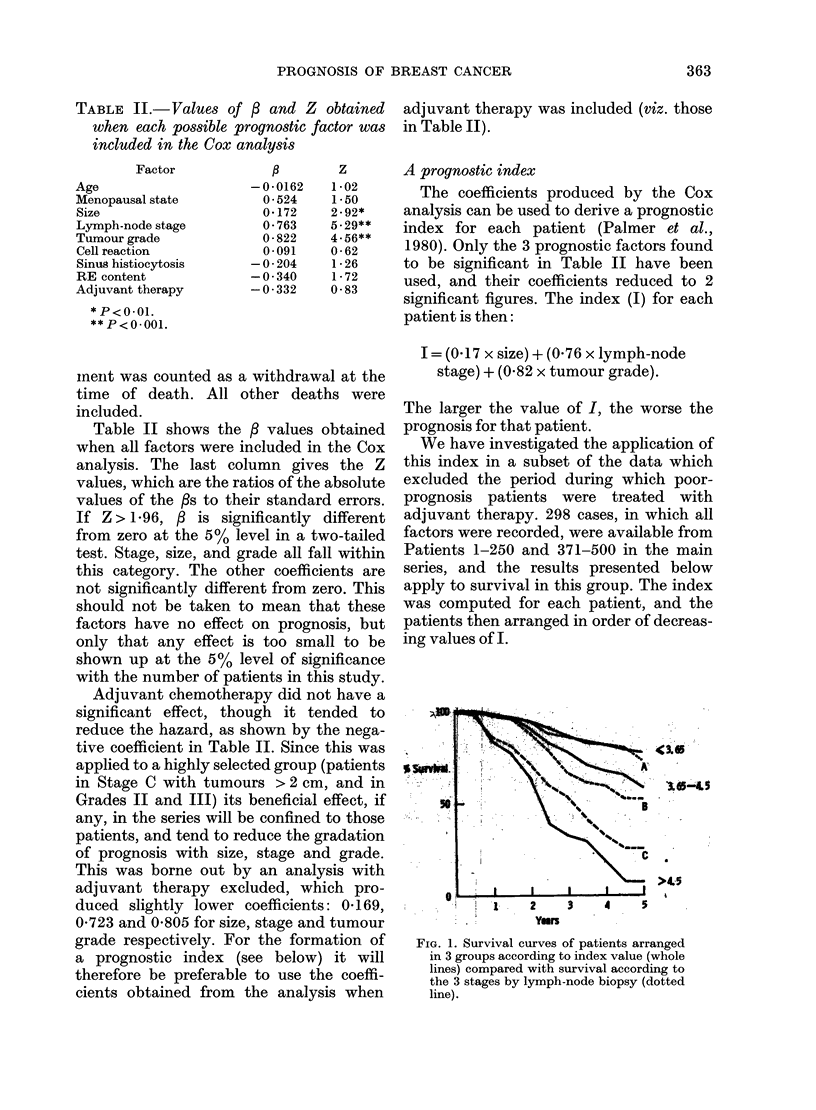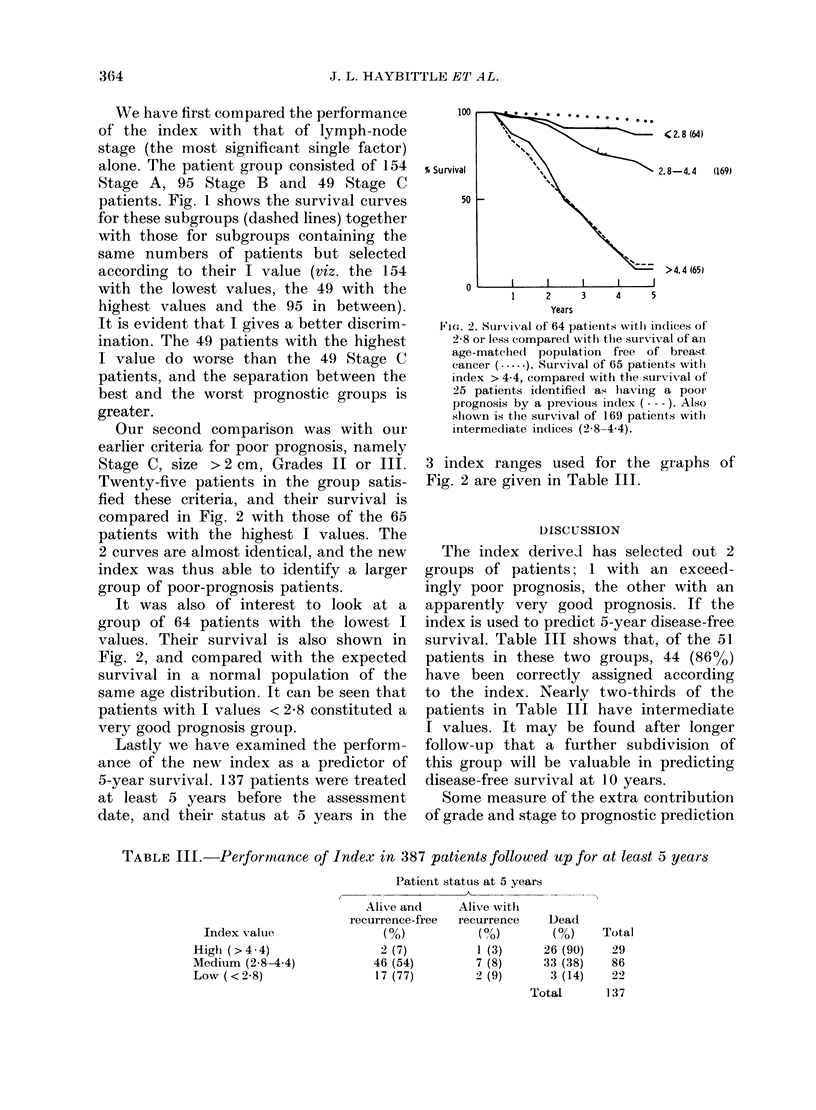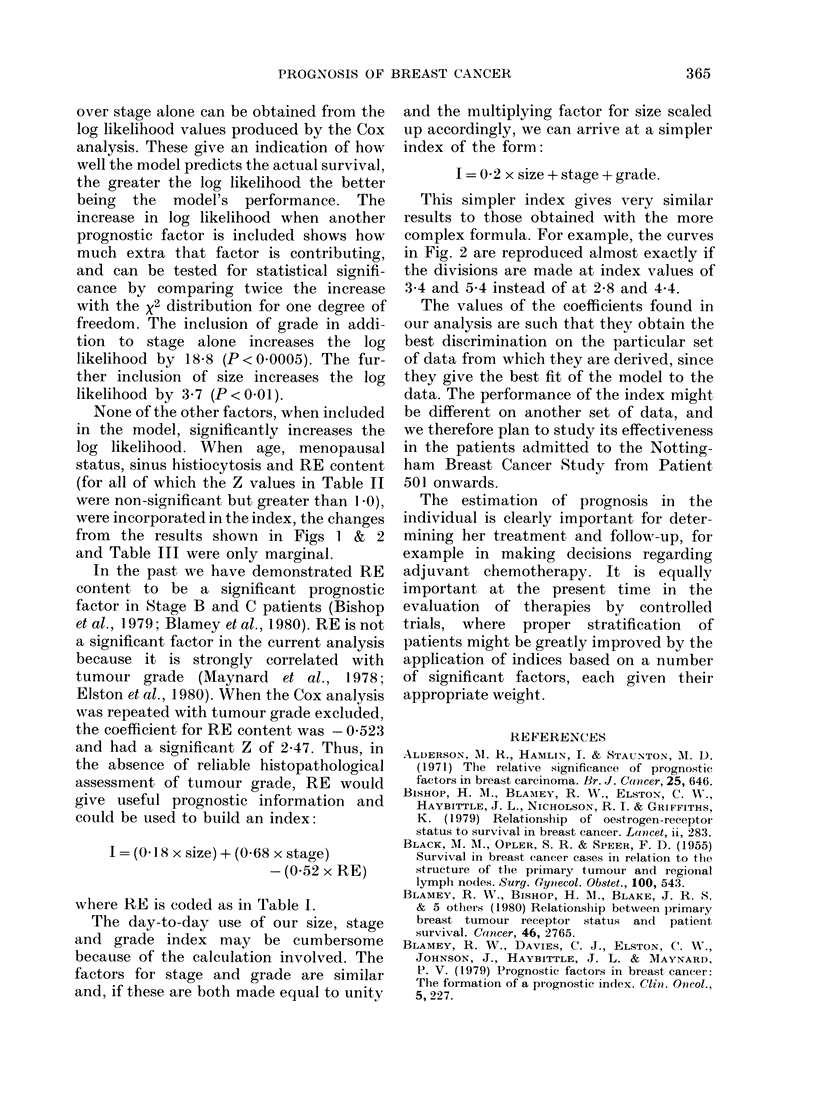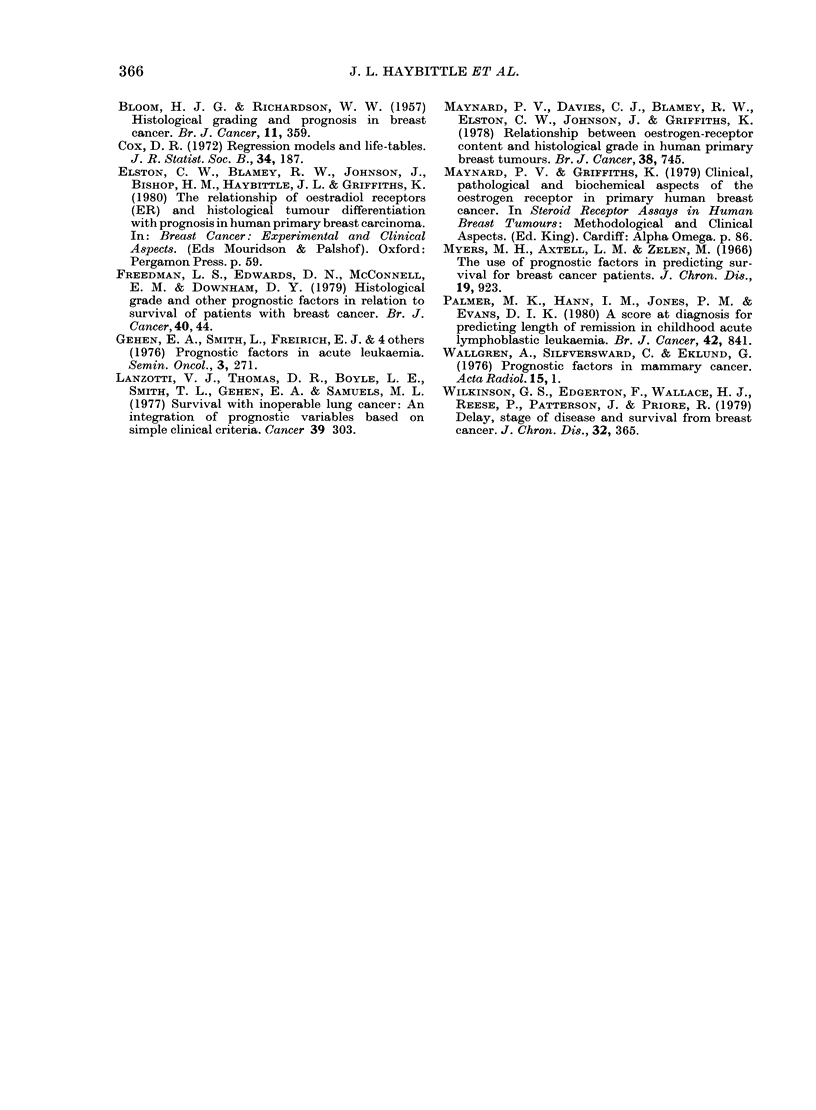# A prognostic index in primary breast cancer.

**DOI:** 10.1038/bjc.1982.62

**Published:** 1982-03

**Authors:** J. L. Haybittle, R. W. Blamey, C. W. Elston, J. Johnson, P. J. Doyle, F. C. Campbell, R. I. Nicholson, K. Griffiths

## Abstract

From a multiple-regression analysis of prognostic factors and survival in a series of 387 patients with primary breast cancer, a prognostic index has been constructed, based on lymph-node stage, tumour size and pathological grade. This index is more discriminating than lymph-node stage alone, and enables a larger group of patients to be identified with a very poor prognosis.


*Br. J. Cancer* (1982) **45**, 361

### A PROGNOSTIC INDEX IN PRIMARY BREAST CANCER

J. L. HAYBITTLE*, R. W. BLAMEY, C. W. ELSTON, J. JOHNSON, P. J. DOYLE, F. C. CAMPBELL, R. I. NICHOLSON† AND K. GRIFFITHS†

*From the City Hospital, Nottingham and the †Tenovus Institute for Cancer Research, Cardiff*

Received 11 September 1981 Accepted 6 November 1981

**Summary.—From a multiple-regression analysis of prognostic factors and survival in a series of 387 patients with primary breast cancer, a prognostic index has been constructed, based on lymph-node stage, tumour size and pathological grade. This index is more discriminating than lymph-node stage alone, and enables a larger group of patients to be identified with a very poor prognosis.**

MANY STUDIES of prognostic factors in breast cancer have been reported in the literature. In some of these, only one factor has been studied in isolation. In others, more than one factor has been investigated by breaking the data down into subgroups, each having the same combination of factors. If more than 3 factors are being studied the number of possible subgroups becomes large and the numbers of patients in each subgroup diminishes correspondingly (Myers *et al.*, 1966). To overcome this problem some form of multivariate analysis can be used to deal with the simultaneous effect of several factors on prognosis (Myers *et al.*, 1966; Freedman *et al.*, 1979; Alderson *et al.*, 1971; Wallgren *et al.*, 1976), and the multiple regression technique described by Cox (1972) has been used in a number of cancer studies (Wilkinson *et al.*, 1979; Gehen *et al.*, 1976; Palmer *et al.*, 1980; Lanzottie *et al.*, 1977). It can make use of all the data from a group of patients having a wide range of follow-up times, and is a powerful technique which makes no assumptions about the form of the survival curve. It has been used to obtain the results reported below.

In the Nottingham Breast Cancer Study, members of a consecutive series of operable patients have all had a number of prognostic factors recorded and have received the same primary treatment. In 1979 we reported preliminary findings in 228 patients who had been followed up for at least 18 months, and we identified by use of stage, size and grade, a group of patients with a very poor prognosis (Blamey *et al.*, 1979). This paper reports the next stage in our attempts to combine factors into a prognostic index.

### PATIENTS AND METHODS

The patients for this study were taken from the first 500 consecutive female patients with primary operable invasive carcinoma of the breast seen and treated, under the care of a single surgeon, by simple mastectomy and triple-node biopsy at the Nottingham City Hospital. The prognostic factors selected for investigation were age, menopausal status (a premenopausal woman being either still menstruating or having a plasma sample containing <50 i.u./l of FSH), tumour size measured in the fresh mastectomy specimen, lymph-node involvement judged by histology, tumour grade, cellular reaction, presence of sinus histiocytosis in lymph nodes, and oestrogen-receptor (RE) content of the primary.

Lymph-node involvement, based on biopsy of a lower axillary node, an apical axillary

* Present address: Physics Department, Addenbrooke's Hospital, Cambridge.
Request for reprints to Professor R. W. Blamey, Department of Surgery, City Hospital, Nottingham.

node and a node from the internal mammary chain, was classified as:

Stage A: Tumour absent from all 3 nodes sampled

Stage B: Tumour in low axillary node only

Stage C: Tumour in apical and/or internal mammary node.

Histological grade (I to III) was determined by a method based on the criteria of Bloom & Richardson (1957). Cellular reaction was scored in 4 categories as described by Black *et al.*, (1955). The absence or presence of sinus histiocytosis in lymph nodes was scored 1 and 2 respectively, whilst cases where all available lymph nodes were completely replaced by tumour were scored zero. RE content was assayed by the method described by Maynard & Griffiths (1979) and tumours were classified as RE+ if they contained >5 fmol specific oestradiol binding per mg cytosol protein.

A group of 387 patients for each of whom all these factors were recorded has been used for the main analysis. The 113 patients excluded were accounted for by 69 with no RE result, 10 with no cell-reaction score, 11 with non-invasive cancer (*i.e.* intra-duct or Paget's), and 23 excluded for a variety of reasons such as previous cancer of the breast, operation not being a simple mastectomy, inadequate clinical details and no follow-up at all.

Following the previous work on a prognostic index (Blamey *et al.*, 1979) a decision was made, after the first 250 patients had been entered in the main study, that patients in the poor-prognosis group (Stage C, size >2 cm, Grade II or III) would be given adjuvant chemotherapy. This policy continued until Patient 370 in the main series, but was discontinued thereafter. Fifteen of the 387 patients were given adjuvant chemotherapy during this period, and it was necessary to take this into account in the analysis.

The first patient in the series was treated just over 6 years before the time of analysis, the last patient just over 1 year before. With this length of follow-up available, it was decided to use survival time as a measure of the outcome of treatment.

To assess the relative importance of the prognostic factors, a series of analyses using the method due to Cox (1972) has been carried out. The simplest use of the method, as reported in this paper, assumes a "proportional hazards" model; *i.e.*, that the relative contribution of each factor to the risk of dying remains constant over the period covered. A more detailed analysis (Freedman & Haybittle, in preparation) using time-dependent variables has shown that in this particular set of data there are no significant departures from such a model.

The Cox method is a multiple regression technique which allows each variable to be evaluated independently, taking into account the effects of all other variables. The coefficients ($\beta$ values) produced by the analysis show how much each factor contributes to the hazard, which is inversely related to survival. A positive value of $\beta$ therefore indicates a poorer survival time as the given variable increases. Table I shows the coding used for the various prognostic factors in our analysis. Survival curves have been calculated using the life-table method with the time divided into 6-monthly intervals.

### RESULTS

These are first presented for the analyses made on the group of 387 patients in whom all the factors were recorded. One patient who died from a road traffic accident without recurrence 4 months after treat-

TABLE I.—*The coding for the various prognostic factors*

| Prognostic factor | Codes used in Cox analysis |
|---|---|
| Age | In years |
| Menopausal state | 0 = premenopausal<br>1 = postmenopausal |
| Size | In cm |
| Lymph-node stage | 1 = A<br>2 = B<br>3 = C |
| Tumour grade | 1 = I<br>2 = II<br>3 = III |
| Cellular reaction | 1 = marked<br>2 = moderate<br>3 = slight<br>4 = none |
| Sinus histiocytosis | 0 = nodes completely replaced by tumour<br>1 = absent<br>2 = present |
| Oestrogen receptor (RE) | 0 = negative<br>1 = positive |
| Adjuvant therapy | 0 = none<br>1 = therapy given |

TABLE II.—*Values of β and Z obtained when each possible prognostic factor was included in the Cox analysis*

| Factor | β | Z |
|---|---|---|
| Age | −0·0162 | 1·02 |
| Menopausal state | 0·524 | 1·50 |
| Size | 0·172 | 2·92* |
| Lymph-node stage | 0·763 | 5·29** |
| Tumour grade | 0·822 | 4·56** |
| Cell reaction | 0·091 | 0·62 |
| Sinus histiocytosis | −0·204 | 1·26 |
| RE content | −0·340 | 1·72 |
| Adjuvant therapy | −0·332 | 0·83 |

* $P<0·01$.
** $P<0·001$.

ment was counted as a withdrawal at the time of death. All other deaths were included.

Table II shows the β values obtained when all factors were included in the Cox analysis. The last column gives the Z values, which are the ratios of the absolute values of the βs to their standard errors. If $Z>1·96$, β is significantly different from zero at the 5% level in a two-tailed test. Stage, size, and grade all fall within this category. The other coefficients are not significantly different from zero. This should not be taken to mean that these factors have no effect on prognosis, but only that any effect is too small to be shown up at the 5% level of significance with the number of patients in this study.

Adjuvant chemotherapy did not have a significant effect, though it tended to reduce the hazard, as shown by the negative coefficient in Table II. Since this was applied to a highly selected group (patients in Stage C with tumours > 2 cm, and in Grades II and III) its beneficial effect, if any, in the series will be confined to those patients, and tend to reduce the gradation of prognosis with size, stage and grade. This was borne out by an analysis with adjuvant therapy excluded, which produced slightly lower coefficients: 0·169, 0·723 and 0·805 for size, stage and tumour grade respectively. For the formation of a prognostic index (see below) it will therefore be preferable to use the coefficients obtained from the analysis when adjuvant therapy was included (*viz.* those in Table II).

*A prognostic index*

The coefficients produced by the Cox analysis can be used to derive a prognostic index for each patient (Palmer *et al.*, 1980). Only the 3 prognostic factors found to be significant in Table II have been used, and their coefficients reduced to 2 significant figures. The index (I) for each patient is then:

$$I=(0·17\times \text{size})+(0·76\times \text{lymph-node stage})+(0·82\times \text{tumour grade}).$$

The larger the value of *I*, the worse the prognosis for that patient.

We have investigated the application of this index in a subset of the data which excluded the period during which poor-prognosis patients were treated with adjuvant therapy. 298 cases, in which all factors were recorded, were available from Patients 1–250 and 371–500 in the main series, and the results presented below apply to survival in this group. The index was computed for each patient, and the patients then arranged in order of decreasing values of I.

FIG. 1. Survival curves of patients arranged in 3 groups according to index value (whole lines) compared with survival according to the 3 stages by lymph-node biopsy (dotted line).

We have first compared the performance of the index with that of lymph-node stage (the most significant single factor) alone. The patient group consisted of 154 Stage A, 95 Stage B and 49 Stage C patients. Fig. 1 shows the survival curves for these subgroups (dashed lines) together with those for subgroups containing the same numbers of patients but selected according to their I value (*viz.* the 154 with the lowest values, the 49 with the highest values and the 95 in between). It is evident that I gives a better discrimination. The 49 patients with the highest I value do worse than the 49 Stage C patients, and the separation between the best and the worst prognostic groups is greater.

Our second comparison was with our earlier criteria for poor prognosis, namely Stage C, size >2 cm, Grades II or III. Twenty-five patients in the group satisfied these criteria, and their survival is compared in Fig. 2 with those of the 65 patients with the highest I values. The 2 curves are almost identical, and the new index was thus able to identify a larger group of poor-prognosis patients.

It was also of interest to look at a group of 64 patients with the lowest I values. Their survival is also shown in Fig. 2, and compared with the expected survival in a normal population of the same age distribution. It can be seen that patients with I values <2·8 constituted a very good prognosis group.

Lastly we have examined the performance of the new index as a predictor of 5-year survival. 137 patients were treated at least 5 years before the assessment date, and their status at 5 years in the 3 index ranges used for the graphs of Fig. 2 are given in Table III.

FIG. 2. Survival of 64 patients with indices of 2·8 or less compared with the survival of an age-matched population free of breast cancer (·····). Survival of 65 patients with index >4·4, compared with the survival of 25 patients identified as having a poor prognosis by a previous index (- - -). Also shown is the survival of 169 patients with intermediate indices (2·8–4·4).

### DISCUSSION

The index derived has selected out 2 groups of patients; 1 with an exceedingly poor prognosis, the other with an apparently very good prognosis. If the index is used to predict 5-year disease-free survival. Table III shows that, of the 51 patients in these two groups, 44 (86%) have been correctly assigned according to the index. Nearly two-thirds of the patients in Table III have intermediate I values. It may be found after longer follow-up that a further subdivision of this group will be valuable in predicting disease-free survival at 10 years.

Some measure of the extra contribution of grade and stage to prognostic prediction

TABLE III.—*Performance of Index in* 387 *patients followed up for at least* 5 *years*

| | Patient status at 5 years | | | |
|---|---|---|---|---|
| Index value | Alive and recurrence-free (%) | Alive with recurrence (%) | Dead (%) | Total |
| High (>4·4) | 2 (7) | 1 (3) | 26 (90) | 29 |
| Medium (2·8–4·4) | 46 (54) | 7 (8) | 33 (38) | 86 |
| Low (<2·8) | 17 (77) | 2 (9) | 3 (14) | 22 |
| | | | Total | 137 |

over stage alone can be obtained from the log likelihood values produced by the Cox analysis. These give an indication of how well the model predicts the actual survival, the greater the log likelihood the better being the model's performance. The increase in log likelihood when another prognostic factor is included shows how much extra that factor is contributing, and can be tested for statistical significance by comparing twice the increase with the $\chi^2$ distribution for one degree of freedom. The inclusion of grade in addition to stage alone increases the log likelihood by 18·8 ($P < 0{\cdot}0005$). The further inclusion of size increases the log likelihood by 3·7 ($P < 0{\cdot}01$).

None of the other factors, when included in the model, significantly increases the log likelihood. When age, menopausal status, sinus histiocytosis and RE content (for all of which the Z values in Table II were non-significant but greater than 1·0), were incorporated in the index, the changes from the results shown in Figs 1 & 2 and Table III were only marginal.

In the past we have demonstrated RE content to be a significant prognostic factor in Stage B and C patients (Bishop *et al.*, 1979; Blamey *et al.*, 1980). RE is not a significant factor in the current analysis because it is strongly correlated with tumour grade (Maynard *et al.*, 1978; Elston *et al.*, 1980). When the Cox analysis was repeated with tumour grade excluded, the coefficient for RE content was $-0{\cdot}523$ and had a significant Z of 2·47. Thus, in the absence of reliable histopathological assessment of tumour grade, RE would give useful prognostic information and could be used to build an index:

$$I = (0{\cdot}18 \times \text{size}) + (0{\cdot}68 \times \text{stage}) - (0{\cdot}52 \times \text{RE})$$

where RE is coded as in Table I.

The day-to-day use of our size, stage and grade index may be cumbersome because of the calculation involved. The factors for stage and grade are similar and, if these are both made equal to unity and the multiplying factor for size scaled up accordingly, we can arrive at a simpler index of the form:

$$I = 0{\cdot}2 \times \text{size} + \text{stage} + \text{grade}.$$

This simpler index gives very similar results to those obtained with the more complex formula. For example, the curves in Fig. 2 are reproduced almost exactly if the divisions are made at index values of 3·4 and 5·4 instead of at 2·8 and 4·4.

The values of the coefficients found in our analysis are such that they obtain the best discrimination on the particular set of data from which they are derived, since they give the best fit of the model to the data. The performance of the index might be different on another set of data, and we therefore plan to study its effectiveness in the patients admitted to the Nottingham Breast Cancer Study from Patient 501 onwards.

The estimation of prognosis in the individual is clearly important for determining her treatment and follow-up, for example in making decisions regarding adjuvant chemotherapy. It is equally important at the present time in the evaluation of therapies by controlled trials, where proper stratification of patients might be greatly improved by the application of indices based on a number of significant factors, each given their appropriate weight.

### REFERENCES

ALDERSON, M. R., HAMLIN, I. & STAUNTON, M. D. (1971) The relative significance of prognostic factors in breast carcinoma. *Br. J. Cancer*, **25**, 646.

BISHOP, H. M., BLAMEY, R. W., ELSTON, C. W., HAYBITTLE, J. L., NICHOLSON, R. I. & GRIFFITHS, K. (1979) Relationship of oestrogen-receptor status to survival in breast cancer. *Lancet*, ii, 283.

BLACK, M. M., OPLER, S. R. & SPEER, F. D. (1955) Survival in breast cancer cases in relation to the structure of the primary tumour and regional lymph nodes. *Surg. Gynecol. Obstet.*, **100**, 543.

BLAMEY, R. W., BISHOP, H. M., BLAKE, J. R. S. & 5 others (1980) Relationship between primary breast tumour receptor status and patient survival. *Cancer*, **46**, 2765.

BLAMEY, R. W., DAVIES, C. J., ELSTON, C. W., JOHNSON, J., HAYBITTLE, J. L. & MAYNARD, P. V. (1979) Prognostic factors in breast cancer: The formation of a prognostic index. *Clin. Oncol.*, **5**, 227.

BLOOM, H. J. G. & RICHARDSON, W. W. (1957) Histological grading and prognosis in breast cancer. *Br. J. Cancer*, **11**, 359.

COX, D. R. (1972) Regression models and life-tables. *J. R. Statist. Soc. B.*, **34**, 187.

ELSTON, C. W., BLAMEY, R. W., JOHNSON, J., BISHOP, H. M., HAYBITTLE, J. L. & GRIFFITHS, K. (1980) The relationship of oestradiol receptors (ER) and histological tumour differentiation with prognosis in human primary breast carcinoma. In: *Breast Cancer: Experimental and Clinical Aspects.* (Eds Mouridson & Palshof). Oxford: Pergamon Press. p. 59.

FREEDMAN, L. S., EDWARDS, D. N., MCCONNELL, E. M. & DOWNHAM, D. Y. (1979) Histological grade and other prognostic factors in relation to survival of patients with breast cancer. *Br. J. Cancer*, **40**, 44.

GEHEN, E. A., SMITH, L., FREIRICH, E. J. & 4 others (1976) Prognostic factors in acute leukaemia. *Semin. Oncol.*, **3**, 271.

LANZOTTI, V. J., THOMAS, D. R., BOYLE, L. E., SMITH, T. L., GEHEN, E. A. & SAMUELS, M. L. (1977) Survival with inoperable lung cancer: An integration of prognostic variables based on simple clinical criteria. *Cancer* **39** 303.

MAYNARD, P. V., DAVIES, C. J., BLAMEY, R. W., ELSTON, C. W., JOHNSON, J. & GRIFFITHS, K. (1978) Relationship between oestrogen-receptor content and histological grade in human primary breast tumours. *Br. J. Cancer*, **38**, 745.

MAYNARD, P. V. & GRIFFITHS, K. (1979) Clinical, pathological and biochemical aspects of the oestrogen receptor in primary human breast cancer. In *Steroid Receptor Assays in Human Breast Tumours*: Methodological and Clinical Aspects. (Ed. King). Cardiff: Alpha Omega. p. 86.

MYERS, M. H., AXTELL, L. M. & ZELEN, M. (1966) The use of prognostic factors in predicting survival for breast cancer patients. *J. Chron. Dis.*, **19**, 923.

PALMER, M. K., HANN, I. M., JONES, P. M. & EVANS, D. I. K. (1980) A score at diagnosis for predicting length of remission in childhood acute lymphoblastic leukaemia. *Br. J. Cancer*, **42**, 841.

WALLGREN, A., SILFVERSWARD, C. & EKLUND, G. (1976) Prognostic factors in mammary cancer. *Acta Radiol.* **15**, 1.

WILKINSON, G. S., EDGERTON, F., WALLACE, H. J., REESE, P., PATTERSON, J. & PRIORE, R. (1979) Delay, stage of disease and survival from breast cancer. *J. Chron. Dis.*, **32**, 365.